# Genome-wide identification and characterization of long non-coding RNAs involved in fruit ripening and the climacteric in *Cucumis melo*

**DOI:** 10.1186/s12870-019-1942-4

**Published:** 2019-08-22

**Authors:** Yunyun Tian, Selinge Bai, Zhenhua Dang, Jinfeng Hao, Jin Zhang, Agula Hasi

**Affiliations:** 1 Key Laboratory of Herbage & Endemic Crop Biotechnology, Ministry of Education, School of Life Sciences, Inner Mongolia University, Hohhot, Inner Mongolia People’s Republic of China; 2 Ministry of Education Key Laboratory of Ecology and Resource Use of the Mongolian Plateau & Inner Mongolia Key Laboratory of Grassland Ecology, School of Ecology and Environment, Inner Mongolia University, Hohhot, Inner Mongolia People’s Republic of China

**Keywords:** Long non-coding RNA, Fruit ripening, Climacteric, RNA-seq, Melon

## Abstract

**Background:**

*Cucumis melo* is a suitable study material for investigation of fruit ripening owing to its climacteric nature. Long non-coding RNAs have been linked to many important biological processes, such as fruit ripening, flowering time regulation, and abiotic stress responses in plants. However, knowledge of the regulatory roles of lncRNAs underlying the ripening process in *C. melo* are largely unknown. In this study the complete transcriptome of *Cucumis melo* L. cv. Hetao fruit at four developmental stages was sequenced and analyzed. The potential role of lncRNAs was predicted based on the function of differentially expressed target genes and correlated genes.

**Results:**

In total, 3857 lncRNAs were assembled and annotated, of which 1601 were differentially expressed between developmental stages. The target genes of these lncRNAs and the regulatory relationship (*cis*- or *trans*-acting) were predicted. The target genes were enriched with GO terms for biological process, such as response to auxin stimulus and hormone biosynthetic process. Enriched KEGG pathways included plant hormone signal transduction and carotenoid biosynthesis. Co-expression network construction showed that LNC_002345 and LNC_000154, which were highly expressed, might co-regulate with mutiple genes associated with auxin signal transduction and acted in the same pathways. We identified lncRNAs (LNC_000987, LNC_000693, LNC_001323, LNC_003610, LNC_001263 and LNC_003380) that were correlated with fruit ripening and the climacteric, and may participate in the regulation of ethylene biosynthesis and metabolism and the ABA signaling pathway. A number of crucial transcription factors, such as *ERFs*, *WRKY70*, *NAC56*, and *NAC72*, may also play important roles in the regulation of fruit ripening in *C. melo*.

**Conclusions:**

Our results predict the regulatory functions of the lncRNAs during melon fruit development and ripening, and 142 highly expressed lncRNAs (average FPKM > 100) were identified. These lncRNAs participate in the regulation of auxin signal transduction, ethylene, sucrose biosynthesis and metabolism, the ABA signaling pathway, and transcription factors, thus regulating fruit development and ripening.

**Electronic supplementary material:**

The online version of this article (10.1186/s12870-019-1942-4) contains supplementary material, which is available to authorized users.

## Background

Genome-wide transcriptome sequencing has revealed that almost 90% of eukaryotic genomes can be transcribed [1], nevertheless only 1–2% of the genome encodes proteins [2]. Thus, non-coding RNAs (ncRNAs) constitute a dominant proportion of the transcriptome. In contrast to protein-coding mRNAs, ncRNAs are characterized by a low level of expression and sequence conservation, thus ncRNAs were originally considered as transcriptional “noise” [3]. With the development of high-throughput sequencing technology and bioinformatic approaches, an increasing number of ncRNAs have been identified and characterized. Two groups of ncRNAs are distinguished on the basis of their length, namely small ncRNAs (sncRNAs) and long ncRNAs (lncRNAs). The sncRNAs are fewer than 200 nucleotides in length and consist of microRNAs and small nucleolar RNAs, and have been well studied in the last decade. In contrast, lncRNAs are longer than 200 nucleotides but lack protein-coding potential [3].

In recent years, it has been reported that lncRNAs may be transcribed from any position of a genome by RNA polymerase II, IV, and V [4–6]. Based on the location and context in the genome, lncRNAs can be classified into five groups, comprising sense lncRNAs, introns originating from natural antisense transcripts, long intergenic non-coding RNAs, intronic ncRNAs, and bidirectional lncRNAs [5]. Previous reports have shown that lncRNAs play important roles in several biological processes, such as gene transcription [7], post-transcriptional modification [8], translation [9], transcriptional interference, protein modification, and regulation of DNA methylation [10, 11]. The best-characterized lncRNAs are mainly from humans and animals, whereas research on plant lncRNAs lags far behind in comparison. Fortunately, the accumulation of plant genetic resources in public databases has stimulated studies of plant lncRNAs. A vast number of lncRNAs have been identified and characterized in plants, such as *Arabidopsis thaliana* [7], *Oryza sativa* [12], *Zea mays* [13], and *Solanum lycopersicum* [14]. For example, about 6480 *A. thaliana* lncRNAs were identified from 200 organ-specific and stress-response transcriptomic data sets [5]. Functional studies have shown that lncRNAs regulate plant growth, development, reproduction, and stress responses [15]. Swiezewski et al. reported a series of cold-induced long antisense RNAs that play a role in the epigenetic silencing of the flowering-time regulatory gene *FLOWERING LOCUS C* in *A. thaliana* [16]. The *WRKY1*-activated lncRNA33732 enhances broad-spectrum resistance to pathogens in tomato [17]. However, knowledge of plant lncRNAs is limited with respect to the diversity and distribution in the genome. Therefore, investigations of lncRNAs on additional plant species that express representative metabolic pathways and/or characteristics are required.

*Cucumis melo*, a member of the Cucurbitaceae family, is an attractive model plant for investigation of important biological processes, including fruit ripening, sex determination, and stress tolerance [18–20]. *Cucumis melo* fruit show notable variation in ripening physiology. The fruit of different varieties can be categorized as climacteric or non-climacteric based on the ripening-related respiration rate [21]. The climacteric is the final physiological stage that marks the beginning of climacteric-dependent fruit ripening, and results in changes in diverse internal and external traits, such as flesh softening, aroma, abscission, rind color [22, 23]. An abrupt increase in ethylene production in the fruit is characteristic of climacteric fruit ripening and usually occurs without an external influence [24]. The physiology and molecular mechanisms of melon fruit ripening have been a research focus in recent years [25]. Guo et al. reported that the rind color changed to yellow from green and flesh firmness increased initially, then declined significantly from 25 days after anthesis (DAA). Coincident with these changes, contents of soluble solids, sucrose, alcohols, and acids were increased in the flesh [26]. Transcriptome profiling of Hami melon fruit development identified 7892 differentially expressed mRNAs (DE-mRNAs), including genes associated with hormone stimulus response, ethylene and sucrose biosynthesis (e.g. sucrose synthases [*SUS*] and 1-amino-cyclopropane-1-carboxylate oxidase [*ACO*]), and several transcription factor families [27]. In addition, our laboratory reported that silencing *CmACO1* postponed fruit ripening of *C. melo* [28]. However, knowledge of the molecular mechanisms underlying melon fruit ripening remains extremely limited.

The melon cultivar used for this work was *Cucumis melo* L. cv. Hetao, white flesh, the peel turned yellow when ripe. With consideration of the hypothesis that lncRNAs might play important roles in melon fruit ripening, in this study we performed RNA sequencing (RNA-seq) to investigate lncRNAs involved in melon fruit development, and predicted the function of lncRNAs based on the position or expression patterns with their target genes. Differential gene expression was characterized among four developmental stages to gain additional insight into the genetic regulation of fruit development. Our results predict the roles of candidate genes and regulatory lncRNAs in fruit development and ripening, and provide a foundation for future studies of the molecular mechanism of ripening in climacteric fruit of melon.

## Results

### Phenotypic data of the *C. melo* fruits

**Fig. 1:**
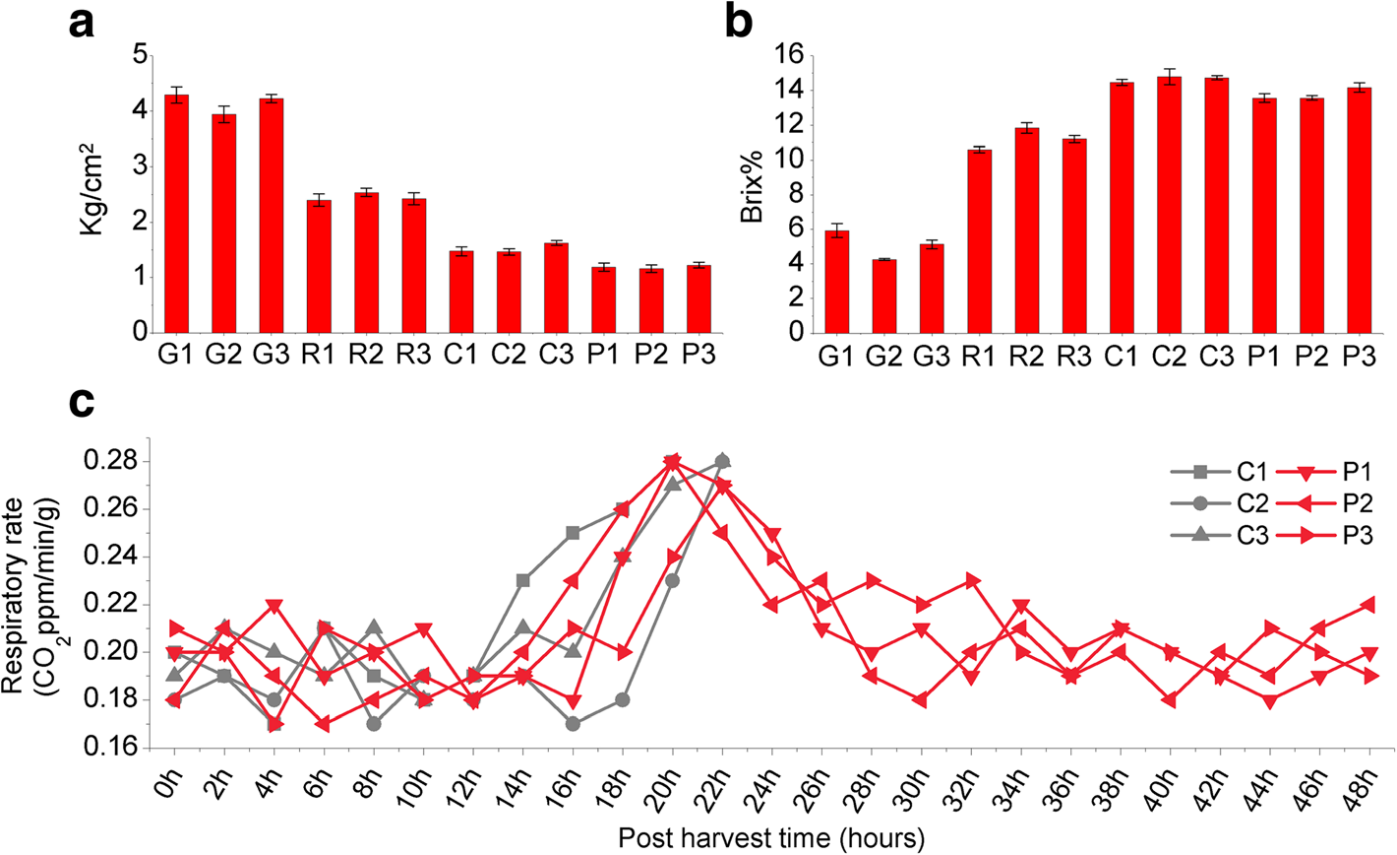
Phenotypic data of the *C. melo* fruits at four stages. **a** Fruit pressure test showed a fast decrease of melon fruit firmness from stage G to P. **b** Soluble solids content progressively increased from stage G to C. **c** Post-harvest respiration rate of melon fruit. The fruit were harvested 36 days after anthesis, and the respiration rate was measured at 2 h intervals for 48 h. Samples from three replicate C stage fruit were collected at 20 h and 22 h after harvest (at these time points the respiration rate curve peaked at 0.25–0.28 CO_2_ ppm/min/g). Samples from P stage fruit were collected after measurement of respiration rate for 48 h. The horizontal axis represents measurement time (hours), and the vertical axis represents the respiration rate (CO_2_ ppm/min/g). Red lines indicate C stage samples, and blue lines represent P stage samples

The fruit pressure test result showed that the fruits turn soft from stage G to P (Fig. 1a). Soluble solids content progressively increased from stage G to C (Fig. 1b). And the respiration rate curve peaked at 0.25–0.28 CO_2_ ppm/min/g (20 h and 22 h after harvest, Fig. 1c).

### RNA sequencing output and genome mapping

In total, 96,820,869 paired-end reads with a mean length of 125 bp were generated in 12 libraries. After filtering out adaptor sequences and low-quality reads, 92,957,247 clean reads were obtained per library. The Q20 and Q30 of the clean reads were greater than 90%, and GC contents of the sequencing outputs were 43–45%. The percentage of clean reads in each library ranged from 92.69 to 96.87%, and approximately 66.39–77.88% of the reads were mapped to the melon reference genome (Table 1).

**Table 1:** Details of the sequencing results for each library

Sample	Raw reads	Clean reads	Q20	Q30	GC content	Total mapped
G1	92829726	89704724	97.52%	93.45%	45.73%	59932101 (69.47%)
G2	97076562	93532184	97.57%	93.58%	45.18%	64011228 (70.75%)
G3	112196528	108293974	97.44%	93.26%	45.31%	63965242 (68.38%)
R1	97152808	94112790	97.58%	93.59%	45.51%	69820488 (77.83%)
R2	104256414	100288582	97.93%	94.46%	43.97%	72182983 (77.17%)
R3	95928048	93646824	97.58%	93.63%	45.52%	84334768 (77.88%)
C1	93075210	86272576	97.4%	93.11%	44.63%	67078059 (72.36%)
C2	93444728	90470954	96.79%	91.79%	44.31%	63906134 (73.3%)
C3	96753370	93541480	97.03%	92.3%	44.35%	62446273 (72.83%)
P1	96824816	92698452	97.4%	93.09%	43.84%	62482447 (66.39%)
P2	92759570	87179606	97.35%	93.01%	44.03%	71196448 (70.99%)
P3	89552648	85744822	97.46%	93.19%	44.32%	65594886 (70.04%)
Reads Average	96820869	92957247.3				

### Characterization of the candidate lncRNAs

**Fig. 2:**
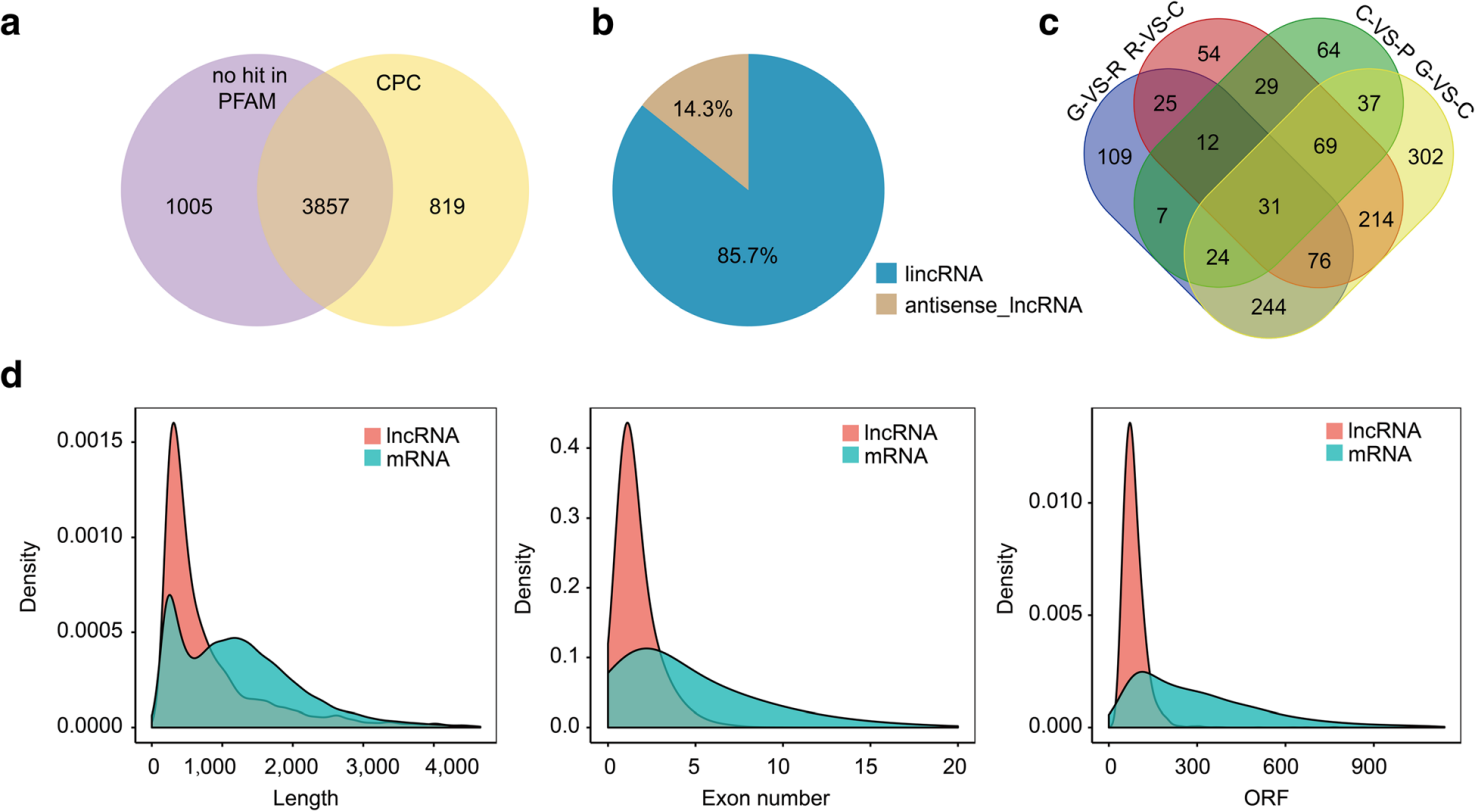
Characterization of candidate lncRNAs and statistical analysis of differentially expressed lncRNAs. **a** Venn diagram of candidate lncRNAs. The coding potential of transcripts was predicted using the CPC tool and the Pfam database. 3857 was the number of lncRNAs that included in CPC but not included in Pfam, 819 was only in CPC, 1005 wasn’t in Pfam or CPC. **b** Classification of the identified lncRNAs; 85.7% of the total lncRNAs were intergenic lncRNAs and 14.3% were anti-sense lncRNAs. **c** Venn diagram of differentially expressed lncRNAs; 528, 510, 76, and 997 differentially expressed lncRNAs were identified among the G-vs-R, R-vs-C, C-vs-P, and G-vs-C stage libraries, respectively. In total, 41 differentially expressed lncRNAs were detected at the four groups. **d** Length distribution of the transcripts. The horizontal axis from left to right indicate the length (left), number of exons (middle), and open reading frame length (right) of the transcripts. The vertical axis represents the density of the transcripts

By matching all of the transcripts using the Coding Potential Calculator (CPC) and the Pfam database, a total of 3857 candidate lncRNAs were predicted (Fig. 2a), which comprised 3307 (85.7%) intergenic lncRNAs and 550 (14.3%) anti-sense lncRNAs (Nature anti-sense transcripts, NATs) (Fig. 2b, Additional file 1). Among the predicted lncRNAs, 142 with mean fragments per kilobase of exons per million mapped fragments (FPKM) > 100 were highly expressed. The overall length of the lncRNAs and their open reading frames were shorter than those of the mRNAs, and the exon number of the lncRNAs was less than that of the mRNAs (Fig. 2d).

### Identification of differentially expressed lncRNAs

On the basis of FPKM values, a total of 1601 differentially expressed lncRNAs (DE-lncRNAs) were identified. The number of DE-lncRNAs at each developmental stage was 528 (G-vs-R), 510 (R-vs-C), 76 (C-vs-P), and 997 (G-vs-C) (Fig. 2c). The cluster analysis showed that the replicates for each developmental stage clustered together. Replicates for the R, C, and P stages formed one group, while G stage replicates formed a separate group (Additional file 11: Figure S1).

### Enrichment analysis of *cis*-acting lncRNAs

After mapping the lncRNAs to the melon reference genome, 3854 lncRNAs were identified close to (< 100 kb) 18,277 protein-coding genes (Additional file 2), named as *cis*-acting lncRNAs. Gene Ontology (GO) enrichment analysis showed that seven, eight, and four GO terms were significantly enriched between stages G and C, G and R, and G and P.

All of the enriched GO terms between the G and C stages were classified under biological process, including response to stimulus (613 genes), response to hormone stimulus (50 genes), response to endogenous stimulus (50 genes), response to auxin stimulus (45 genes), hormone biosynthetic process (15 genes), pheromone metabolic process (15 genes), and pheromone biosynthetic process (15 genes; Additional file 3). The Kyoto Encyclopedia of Genes and Genomes (KEGG) analysis showed that the target genes of lncRNAs were enriched in 119 KEGG pathways. Enriched pathways associated with fruit development included plant hormone signal transduction, carotenoid biosynthesis, carbon metabolism, and carbon fixation in photosynthetic organisms (Additional file 4).

### Enriched *cis*-target genes in plant hormone signal transduction

A total of 171 DE-mRNAs were involved in plant hormone signal transduction. Of these DE-mRNAs, 139 co-located with 250 lncRNAs (Additional file 5), and 109 of the lncRNAs were differentially expressed. Among these DE-lncRNAs, LNC_003452 was the most highly expressed and co-located with 43 mRNAs. *CmACCO3* was a target mRNA of LNC_003452, and located 94,591 bp upstream of the lncRNA.

### Enrichment analysis of co-expressed genes of lncRNAs

The potential correlated pairs of lncRNAs and mRNAs were predicted using co-expression analysis. A total of 245,368 interactions between 2258 lncRNAs and 11,102 protein-coding transcripts were detected. Among all the interactions, 245,100 were *trans*-acting (distance beyond 100 kb, Additional file 2). Functional analysis showed that the co-expressed genes were enriched in 3788 GO terms. Among the GO terms, 2127, 1170, and 491 were classified under biological process, molecular function, and cellular component, respectively (Additional file 6). Interestingly, some of the GO terms were closely associated with fruit development, including response to hormone stimulus, response to auxin stimulus, and hormone biosynthetic process. The co-expressed genes were enriched in 120 KEGG pathways (Top 20 enriched pathways of each group showed in Additional file 7). Among the DE-mRNAs in any pairwise comparison, 171 were involved in plant hormone signal transduction pathways, of which 130 were co-expressed with 422 DE-lncRNAs. In the carotenoid biosynthesis pathway, 18 DE-mRNAs were co-expressed with 204 DE-lncRNAs.

### Systematic cluster analysis, co-expression network construction, and PPI analysis

**Fig. 3:**
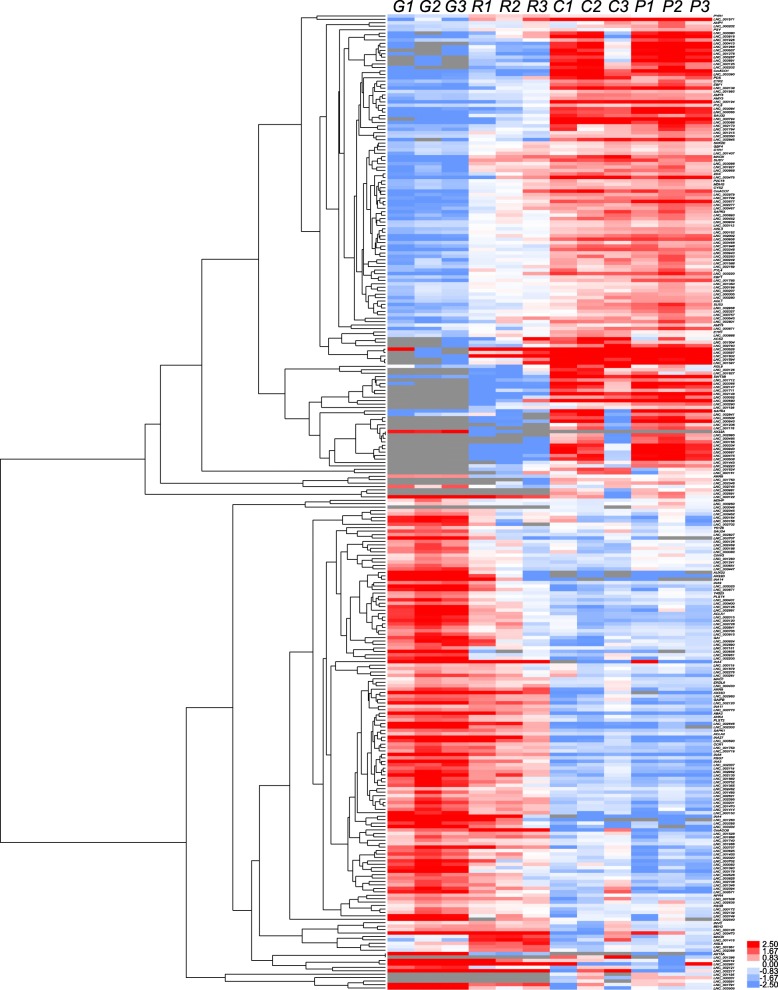
Systematic cluster analysis of DE-genes (fold change > 2) and their co-expressed DE-lncRNAs. The DE-genes and the DE-lncRNAs showed similar expression changes, and stages G and R formed one group, and stages C and P formed a second group. The red to lilac color gradient indicates a high to low level of expression. Target genes correlated with the functions of response to hormone stimulus, carotenoid or ethylene biosynthesis, and sugar and main organic acid metabolism

**Fig. 4:**
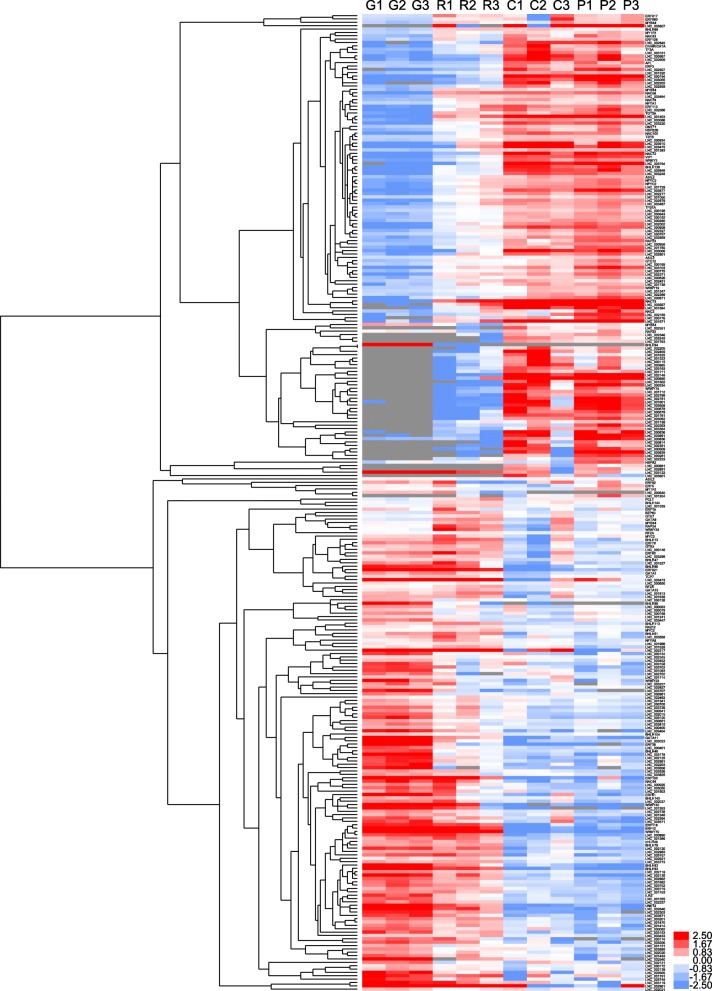
Systematic cluster analysis of DE-TFs and their co-expressed lncRNAs, and stages G and R formed one group, and stages C and P formed a second group

To gain insight into the temporal and spatial transcription patterns and putative functions between the lncRNAs and their co-expressed mRNAs during *C. melo* fruit development, 83 target genes, 91 DE-TFs (fold change > 2), and their co-expressed DE-lncRNAs were selected and a systematic cluster analysis was performed. Interestingly, the expression patterns were consistent and for each data set two groups were resolved (stages G and R, and stages C and P; Figs. 3 and 4), which indicates a correlation with development and the respiratory climacteric process in *C. melo* fruit. Network analysis showed that one lncRNA could co-regulated with multiple mRNAs and vice versa, suggesting complex interactions between lncRNAs and mRNAs. The protein interactions (PPI) analysis showed that *CmACO1* and *Sucrose Synthase Y* (*SUSY*) interacted with *ACS1* and *Agamous-like 2* (*AGL2*), respectively. *Serine/threonine-protein kinase 3* (*CmSAPK3*) and *CmSAPK4* both interacted with *CmACC1*.

### Expression profiling of the key elements associated with *Cucumis melo* fruit ripening

**Fig. 5:**
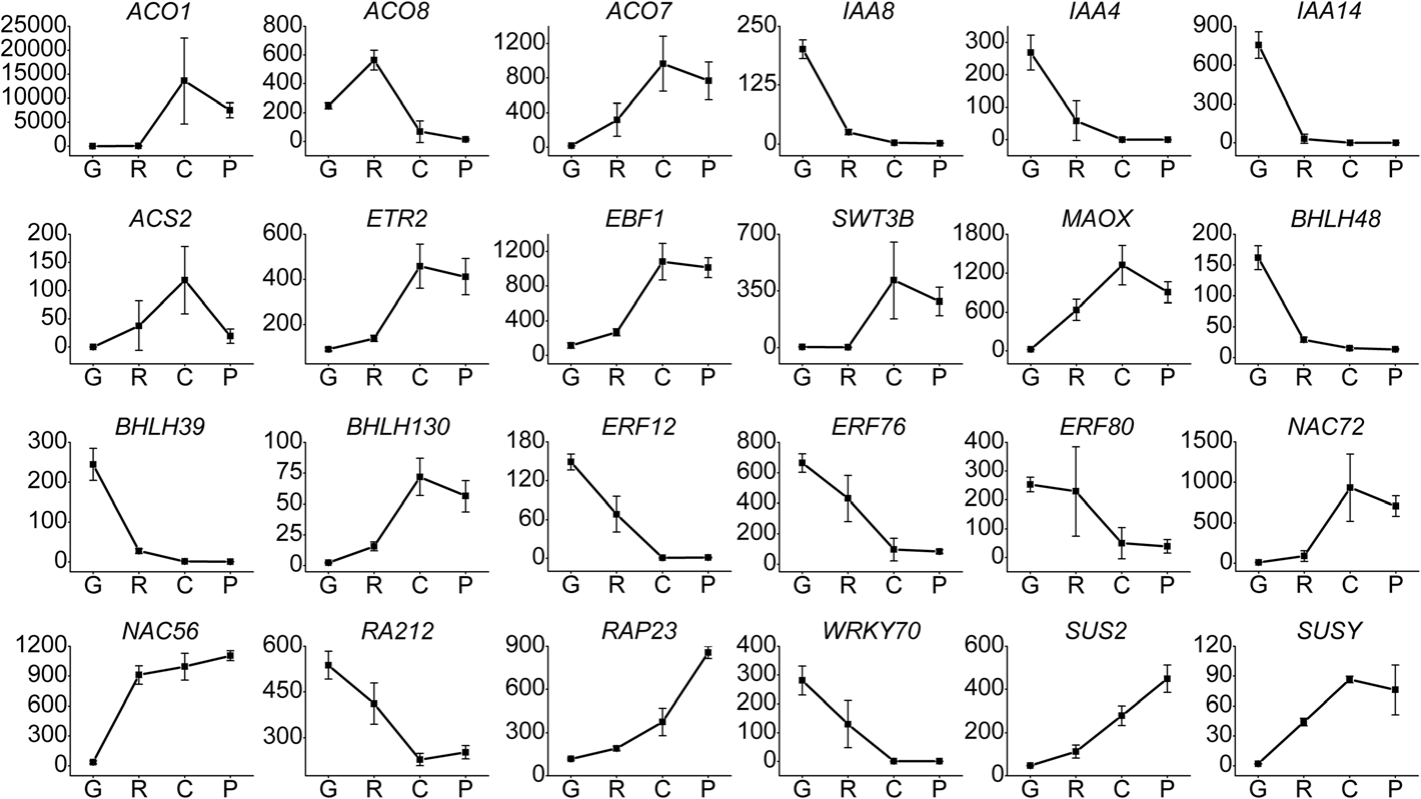
Expression patterns of genes associated with *C. melo* fruit development. The horizontal axis represents the four developmental stages of *C. melo* fruit. The vertical axis represents the average FPKM value of mRNAs in four samples

**Fig. 6:**
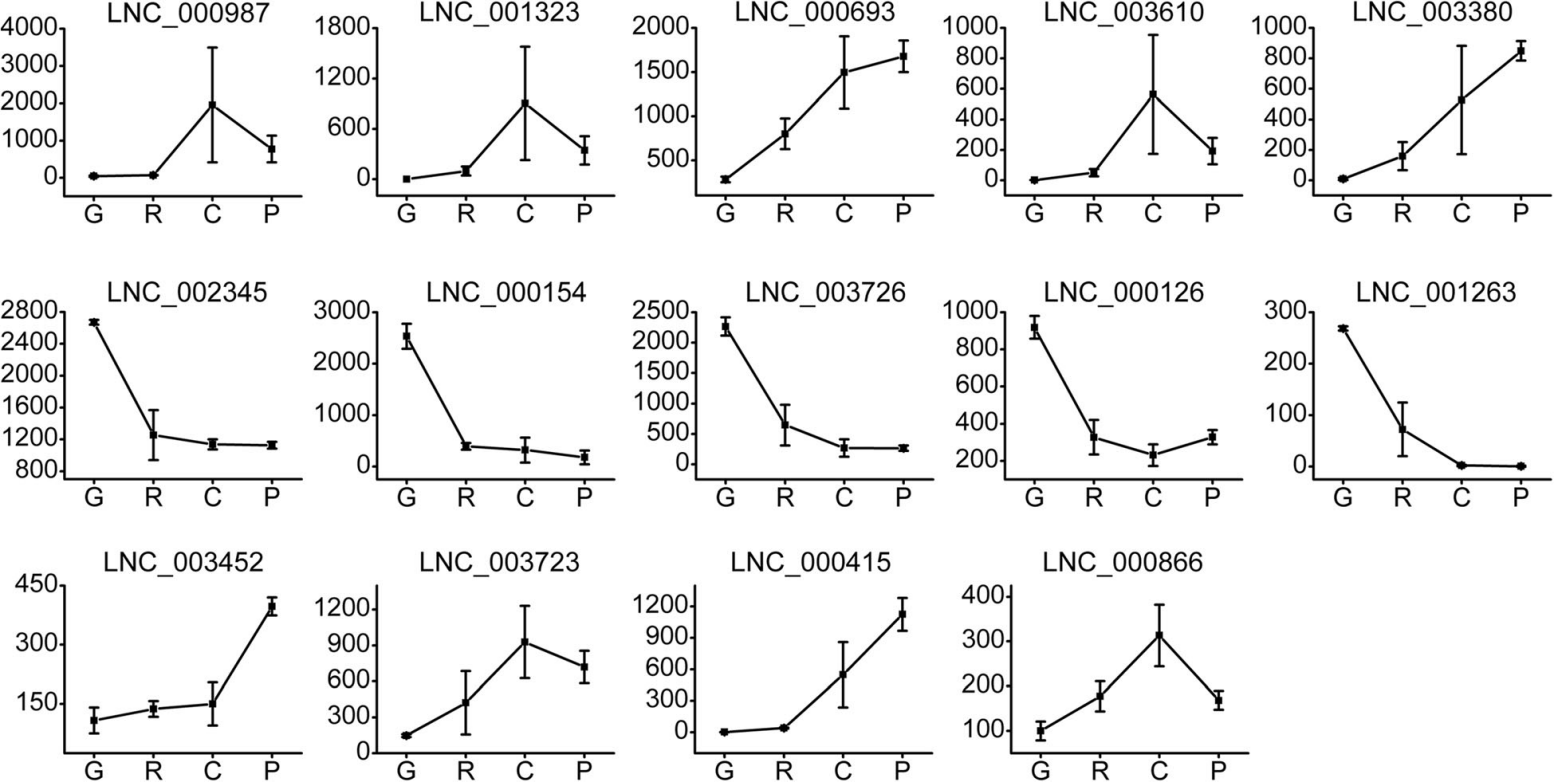
Expression patterns of lncRNAs associated with *C. melo* fruit development. The horizontal axis represents the four developmental stages of *C. melo* fruit. The vertical axis represents the average FPKM value of lncRNAs in four samples

**Fig. 7:**
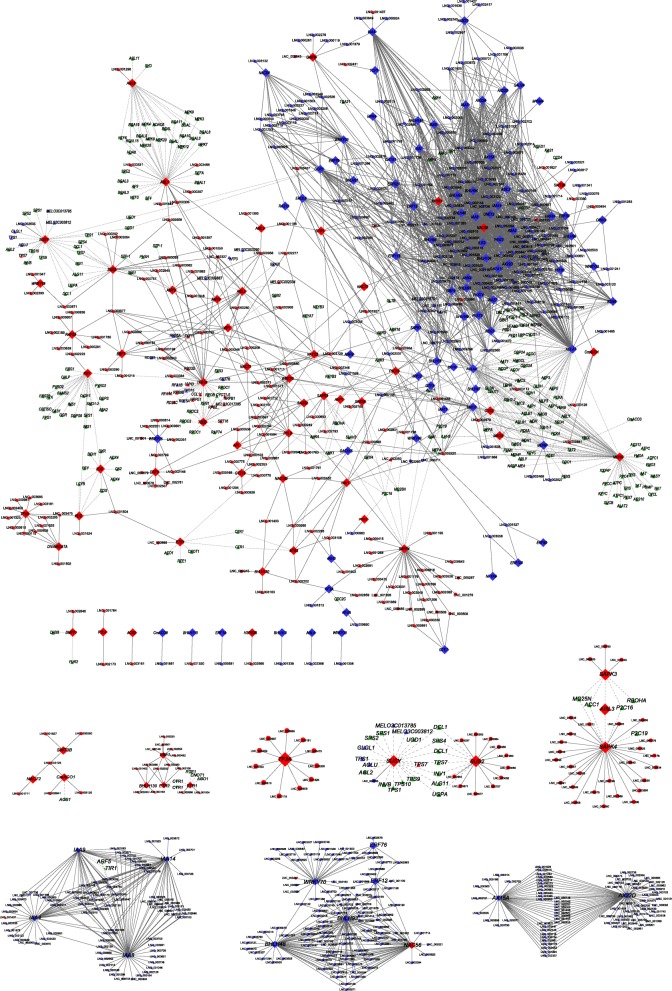
Co-expression networks among target genes and co-expressed lncRNAs. The target genes were correlated with the functions of response to hormone stimulus, carotenoid or ethylene biosynthesis, sugar and main organic acid metabolism, and also DE-TFs fold change > 2. The correlation between lncRNAs and mRNAs is represented by solid lines, and protein–protein interactions are represented as dotted lines. The subnetworks were extracted from the total networks. Diamonds, circles, and green triangles represent target genes, lncRNAs, and genes that interacted with target genes, respectively. Red and blue symbols represent transcripts that were up- and down-regulated between G and C group, respectively

Genes that play important roles in pathways including hormone stimulus, carotenoid or ethylene biosynthesis, and sugar and main organic acid metabolism were selected and their expression profiles were analyzed. *CmACO1*, *MAOX* (similar to NADP-dependent malic enzyme), *EBF1*, *CmACO7*, *ETR2*, and *SWT3B* had the highest expression levels at stage C. The average FPKM of *CmACO1* was extremely high (almost 13,613), followed by *MAOX*, *EBF1*, *CmACO7*, *ETR2*, and *SWT3B*, with average FPKM values of 1323.5, 1082, 965.77, 457.7, and 416, respectively (Fig. 5). LNC_000987, LNC_001323 and LNC_003610showed similar expression patterns at stage C, and their average FPKM values were 1954.4, 901.0 and 563.0 respectively. LNC_000693 and LNC_003380 were also highly expressed at stage C, and increased from stage G to P (Fig. 6). *IAA14*, LNC_002345, LNC_000154, LNC_003726, and LNC_000126 showed the highest expression levels at stage G, and had mean FPKM values of 954, 2670.6, 2533.8, 2266, and 919, respectively. *SUS2* and *SUSY* (sucrose synthase) were up-regulated from stage G to P, and 11 DE-lncRNAs were co-expressed with *SUS2*. The correlations between the DE-mRNAs and lncRNAs described above are shown in the co-expression network (Fig. 7).

Among the analyzed DE-TFs, *bHLH130* was significantly up-regulated from stage R to C (fold change > 4.5), and LNC_003066 was co-expressed with this DE-TF. *bHLH48* and *bHLH93* were highly expressed at stage G, then significantly down-regulated at stage R. The average FPKM value of *NAC72* was 933 at stage C. *NAC56* was highly expressed at stages R, C, and P, but the co-expressed lncRNAs were all negatively correlated (Fig. 7). *WRKY70* was highly expressed at stages G and R, then significantly down-regulated at stage C. Several co-expressed lncRNAs of *WRKY70* were identified, and all the correlations were positive except for one (LNC_003467). Among them, LNC_001263 was down-regulated from the G to C stages, and the FPKM value decreased from 269 to 2. *ERF12*, *ERF76*, and *ERF80* were significantly down-regulated from stages G to C (Fig. 5). The gene id of the genes included were provided in Additional file 8.

### Validation of differentially expressed lncRNAs

The expression patterns of 10 lncRNAs with only one transcript were validated at the four developmental stages by qRT-PCR. The results confirmed that the expression patterns of the selected lncRNAs were consistent with the expression levels calculated from the RNA-seq data (Additional file 12: Figure S2). The results showed that the pipeline used in the present study was extremely strict in identifying putative lncRNAs, and suggested that the identified lncRNAs were genuinely expressed.

## Discussion

### An informative dataset of lncRNAs associated with fruit ripening in *Cucumis melo*

Increasingly, lncRNAs are recognized as an important class of regulatory molecules, but they remain poorly studied in model plants. Plant lncRNAs are linked to biological processes such as gene silencing, flowering-time regulation, abiotic stress response, and numerous other developmental pathways [29]. However, in *C. melo* fruit, no information on lncRNAs was available. In the current study, by applying RNA-seq and genome mapping approaches, 3857 lncRNAs were identified at four stages of *C. melo* fruit development, of which 1601 lncRNAs were differentially expressed. Of all pairwise comparisons among the four analyzed stages, stages G and P had the highest number of DE-lncRNAs (1184), suggesting that gene expression regulatory patterns changed significantly in the process of *C. melo* fruit development. In addition, 510 DE-lncRNAs between stages R and C were identified, of which some might be closely associated with molecular regulation of the respiratory climacteric. Based on the physical location and expression relationships between lncRNAs and their target mRNAs, further prediction of the *cis* or *trans* regulatory activity of the identified lncRNAs was performed. As a result, 3854 lncRNAs were identified as close to 18,277 protein-coding genes. A total of 245,368 interactions between 2258 lncRNAs and 11,102 co-expression genes, and 245,100 were *trans*-acting. Finally, we analyzed the functions of DE-lncRNAs based on their target genes and the co-expression genes, of which some were involved in diverse processes associated with fruit development, including response to hormone stimulus, carotenoid or ethylene biosynthesis, and sugar and main organic acid metabolism. The present results predicted the regulatory function of the lncRNAs during *C. melo* fruit development and ripening, and lay a foundation for further detailed studies.

### *Cis*/*trans* roles of the lncRNAs and their target DE-genes involved in plant hormone signal transduction

Fruits are important resources for human and animal nutrition owing to their high vitamin, mineral, and sugar contents. In addition, fruits are rich sources of antioxidant compounds, such as carotenoids, anthocyanins, and flavonoids [30], which are of great economic value. Ripening is crucial for development of the flavor and nutritional quality of fruits [31]. Fruit ripening is a developmental process coordinated by complex networks of interacting genes and signaling pathways. Plant hormones play important roles in the regulation of this process. In fruit trees and crops, a variety of genes and regulators encode and/or regulate enzymes in the plant hormone signaling transduction pathways of fruit components during ripening. For example, in tomato, the MADS-box genes ripening inhibitor (*RIN*) and agamous-like 1 (*AGL1*) dramatically affect fruit ripening [28]. In tomato, pepper, banana, and strawberry, the dominant endogenous auxin indole-3-acetic acid (IAA) defers early fruit development [24]. However, current knowledge of the roles of hormones (except for ethylene) in the development and ripening of climacteric and non-climacteric fruits is limited, therefore the genetic and molecular factors involved in the plant hormone signal transduction pathway that regulate ripening should be identified and characterized. One of the main objectives in the present study was to understand how the co-expression network among the lncRNAs and mRNAs controls development of melon fruit. As a result, we identified 109 DE-lncRNAs co-located with the DE-mRNAs involved in plant hormone signal transduction, and 422 DE-lncRNAs co-expressed with the DE-mRNAs involved in the same process.

Auxin plays a primary role during the conversion of the ovary into a growing fruit and controls many aspects of fruit development, including fruit set and growth, ripening and abscission [32]. In auxin transduction, auxin-induced proteins and IAA are the most well-studied components and are encoded by *Auxin-Induced* (*AX*) genes and *IAA*. In the current study, 22 auxin transduction-related DE-genes were all co-expressed with LNC_002345, including *IAA4*, *IAA8*, *IAA9*, *IAA14*, *AX22D*, and *AX15A*, and 16 DE-genes were co-expressed with LNC_000154, including *AX22D*, *AX15A*, *IAA8*, and *GID1C* (a gibberellin receptor) (Fig. 6). These genes were all highly expressed at stage G, then significantly down-regulated at stage R. However, LNC_002345 was highly expressed at all four stages and the average FPKM value was 2670.6 at stage G, then was down-regulated by more than two-times at stage R and its expression remained stable until stage P. The FPKM value of LNC_000154 at stage G was 2533.8, then the gene was significantly down-regulated to 395 at stage R. We predicted that LNC_002345 and LNC_000154 might regulate the expression of genes associated with auxin signal transduction, and thereby be involved in fruit development. Other highly expressed lncRNAs that also interacted with auxin signal transduction genes are shown in Fig. 7.

With regard to the abscisic acid (ABA) signaling pathway, ABA promotes strawberry fruit ripening, and the ABA content peaks earlier than that of ethylene, which suggests that ABA and ethylene both promote strawberry fruit maturation [33]. *SAPK* genes encode serine/threonine-protein kinase and play a role in the ABA signaling pathway. Among the present findings, the most highly expressed lncRNA at stage C was LNC_000693, which was co-expressed with *CmSAPK3*. LNC_003380 and LNC_000415 were up-regulated from stage G to stage P (average FPKM values increased from 8.7 to 849, and 0.5 to 1124, respectively), and all three lncRNAs were co-expressed with *CmSAPK4*. The expression patterns and interactions between lncRNAs and *SAPK* genes of the above-mentioned three lncRNAs suggest that they might regulate *C. melo* fruit ripening by participating in the ABA signaling pathway. PPI analysis showed that *CmSAPK3* and *CmSAPK4* both interacted with *CmACC1* (which encodes an ethylene precursor), implying that *SAPK* genes may be associated with ethylene biosynthesis in *C. melo* fruit.

### lncRNAs and their co-expressed genes associated with ethylene and sucrose biosynthesis and signal transduction

Fleshy fruits can be classified into two physiological groups, climacteric and non-climacteric, according to their respiration characteristics and rate of ethylene release during ripening. Ethylene synthesis in climacteric fruits, such as tomato, apple, banana, and *C. melo*, is essential for the normal fruit ripening process [24]. In the present study, *CmACO1* and *CmACO7* were significantly up-regulated at the post-ripening stage P, which was consistent with previous reports [24]. Mutation of *ETR1–1* results in disruption of ethylene binding during the ethylene response in *Arabidopsis* [34]. In *C. melo*, *EBF1*, *ETR1*, and *ETR2* were up-regulated during fruit development. We predicted that *ETR1* might interact with LNC_000866 and LNC_001504. The average FPKM value of LNC_000866 from stage G to C increased from 99.8 to 313, then was down-regulated to 168 at stage P. *ETR2* might interact with LNC_003066 in the same pathway. These results revealed that lncRNAs may interact with *ETR1/2* and play roles in ethylene signaling transduction in *C. melo*.

Recent studies have acknowledged that sucrose acts as a signal to modulate a wide range of processes in plants, including fruit development. Abdullah et al. detected four genes of the *SUS* family that were significantly up-regulated during fruit development of Chinese pear [35]. In the present study, *SUS2* and *SUSY* (which encode sucrose synthases) showed similar expression patterns from stages G to P, which was consistent with the physiological data (soluble solids content test). In addition, the expression levels of *CmMAOX* (NADP-dependent malic enzyme) and *CmSWT3B* (bidirectional sugar transporter) increased by 52 and 75 times, respectively, from stages G to C, and then were down-regulated at stage P. The changes in expression of these two genes suggest that they may perform regulatory roles during fruit development in *C. melo*, but the specific functions of the two genes have not been elucidated and require investigation in the near future.

### Expression profiling and regulatory role of transcription factors and their co-expressed lncRNAs in *Cucumis melo*

Transcription factors (TFs) are regulatory proteins responsible for the transcriptional activation or repression of target genes by recognizing specific, short DNA sequences (6–12 bp) in their regulatory regions. Studies on the regulatory mechanisms and functions of TFs associated with fruit development have increasingly been considered as research hotspots. However, current knowledge is extremely limited and is mainly confined to several TF families, such as *ERF*, *RIN*, and *MADS-box* [36], thus an improved understanding of the roles of TFs in the process of fruit ripening is needed.

In the present study, we analyzed the DE-TFs during *C. melo* fruit ripening, and determined that the TFs differentially expressed among the four developmental stages were largely distributed in the *bHLH*, *ERF*, *NAC*, and *WRKY* TF families. The present results were consistent with observations reported for tomato fruit ripening [37], suggesting that the transcriptional strategies during ripening in *C. melo* may be similar with other climacteric fruit. The systematic cluster analysis showed that the expression patterns of DE-TFs (fold change > 2) and their co-expressed DE-lncRNAs are consistent (Fig. 4), which indicated that these DE-TFs may be associated with regulation of the respiratory climacteric in *C. melo*.

The *NAC* gene family is considered to be involved in the regulation of fruit ripening. NAC protein can bind to the promoter of *ACS* and *ACO* in ripening peach fruit tissues [38]. *NAC4* has been confirmed to play a role in tomato fruit ripening [39]. Ríos et al. identified *NAC56* as a fruit ripening quantitative trait locus that regulates climacteric ripening in melon [40]*.* In the present investigation, *NAC56* was significantly up-regulated from stages G to R, which was consistent with the expected results, thus the gene may play a role in regulation of fruit ripening in *C. melo*. In addition, *NAC72* was remarkably up-regulated from stages R to C, then down-regulated slightly at stage P. We speculate that *NAC72* might play a crucial role in the *C. melo* climacteric process. The *bHLH* gene family is a common TF family among plants. *MdbHLH93* interacts directly with an ABA-responsive protein, *MdBT2*, and thus delays leaf senescence [41]. In *C. melo*, *bHLH130* was significantly up-regulated from stages R to C (fold change > 4.5), and LNC_003066 was co-expressed with *bHLH130*. LNC_003066 and *bHLH130* might function as positive regulators during *C. melo* fruit ripening. *bHLH48* and *bHLH93* were highly expressed at stage G, then were down-regulated significantly at stage R; in addition, we detected that LNC_000126 was co-expressed with *bHLH93*. Whether LNC_000126 and *bHLH93* are negative regulators of *C. melo* fruit ripening requires further study. *WRKY* family genes are generally considered to be stress tolerance regulators, but recent studies implicate *WRKY* TFs in fruit development. It has been confirmed that the *WRKY* domain facilitates binding of the protein to the SURE (sugar-responsive *cis*-element) element in the promoter regions of target genes [42]. Zhou et al. predicted that *FvWRKY* genes may operate in the ABA, IAA, and sucrose signaling network during strawberry fruit development [43]. We determined that the average FPKM value of *WRKY70* was 281 at stage G, and was down-regulated to 130 at stage R and to 0.65 at stage C. Among the DE-lncRNAs that were co-expressed with *WRKY70*, only LNC_003467 were negatively correlated, LNC_001263 was highly expressed at stage G and was significantly down-regulated at stage C (Fig. 6). We speculated that *WRKY70* might be a negative regulatory factor in the climacteric process, and that it might interact with LNC_001263. LNC_000987, LNC_001323, and LNC_003610 were up-regulated and highly expressed at stage C, and were co-expressed with *TF3A*, which participates in nucleic acid binding. As an important plant-specific TF, the *AP2/ERF* gene family plays crucial roles in plant growth, development, and stress responses. *ERF* genes mediate transcription of ethylene-regulated genes. The *AP2/ERF* family has been widely studied in many plant species, such as *Arabidopsis thaliana* [44] and *Vitis vinifera* [45]. Among the DE-*ERFs* that we detected, *ERF12*, *ERF76*, and *ERF80* were highly expressed at stages G and R, and were significantly down-regulated at stage C. Thus, ethylene-responsive genes may not be induced by these *ERF* genes and a portion of the ethylene synthesized may not be consumed in stage C. The concurrent significant up-regulation of *CmACO1* and *CmACO7* would notably promote ethylene biosynthesis. These findings suggested that the climacteric in *C. melo* might depend on the synthesis of a large amount of ethylene as well as reduction in consumption of ethylene.

## Conclusions

The climacteric is a crucial physiological process during ripening of *C. melo* fruit. By performing high-throughput RNA sequencing and genome mapping, we constructed an informative dataset of lncRNAs and differentially expressed mRNAs associated with *C. melo* fruit ripening. The DE-lncRNAs and DE-mRNAs involved in plant hormone signal transduction, ethylene biosynthesis, sucrose biosynthesis and several transcription factors were investigated. Among these processes and metabolic pathways, the expression patterns of genes (*CmACO*, *CmSAPK*, *CmETR*), lncRNAs (LNC_000987, LNC_000693, LNC_001323, LNC_003610 and LNC_003380) and transcription factors (*ERFs*, *NAC56*, *NAC73*, and *WRKY70*) suggest that they may be closely associated with fruit ripening and the climacteric in *C. melo*. The biosynthesis and consumption of ethylene is likely to be a key element in the ripening process. The present results provide insight into the molecular mechanism of the respiratory climacteric, and lay a foundation for future studies of the ripening process in climacteric fruit of *C. melo*.

## Methods

### Plant material, RNA extraction and transcriptome sequencing

*C. melo* (Cultivar. Hetao) plants were grown in open experimental field (Bayan Nur, Inner-mongolia) from May to August in 2016 (6 °C–34 °C), temperature difference between day and night were more than 10 °C, self-pollination strictly. To identify expressed lncRNAs of *Cucumis melo* fruit, samples were collected at four developmental stages, namely growing (G), ripening (R), climacteric (C), and post-climacteric (P), with three biological replicates per stage. The G and R stage samples were harvested at 18 and 36 DAA, respectively. The collection of samples from harvested C stage fruit coincided with the peak respiration rate at 20 and 22 h post-harvest. The P stage samples were collected from post-climacteric harvested fruit at 48 h post-harvest. The fruit pressure was tested using fruit pressure tester (EFFEGIDI, FT 011, Italy). Four symmetry points were measured at the vertical section of the mesocarp. And soluble solids content was tested strictly following the instructions (Pocket refractometer: ATAGO, Cat. No. 3830, Japan). Respiration rate was also measured using a Fruit and Vegetable Respiratory Meter (3051H, TOP Instrument Co., Zhejiang, China). The time course of changes in the respiration rate of fruit harvested at 36 DAA.

After harvesting, the pericarp was immediately dissected, the flesh was frozen in liquid nitrogen, and stored at − 80 °C. Total RNA was isolated using the TRIzon Reagent (Kangwei Biotech, Beijing, China) in accordance with the manufacturer’s instructions. Genomic DNA was removed from extracted total RNA by treatment with DNase I (RNase-free; Takara Bio, Shiga, Japan). The RNA integrity, quality, and quantity were checked after 1% agarose gel electrophoresis with a NanoPhotometer® spectrophotometer (IMPLEN, Westlake Village, CA, USA) and using the Nano 6000 Assay Kit with the Agilent 2100 Bioanalyzer™ system (Agilent Technologies, Santa Clara, CA, USA), respectively. Finally, 12 libraries were constructed and sequenced on an Illumina HiSeq 2500 platform.

### Data filtering and genome mapping

Clean reads were obtained by removing reads containing adapter and poly-N sequences. Data processing of raw reads was quality checked trimmed for low-quality bases and adaptors by using Illumiprocessor Version 2.0 (https://illumiprocessor.readthedocs.io/en/latest/). The Q20 and Q30 percentages and GC content of the clean data were calculated. All downstream analyses were based on the clean data consisting of high-quality reads. Reference genome and gene model annotation files were downloaded from the Melonomics website (Genome/Melon_genome_v3.5.1, http://melonomics.cragenomica.es/). An index of the reference genome was built using Bowtie (Version 2.0.6) and paired-end clean reads were aligned to the reference genome using TopHat (Version 2.0.9, *N* = 2, library type = fr-first strand).

### lncRNAs library construction and gene expression quantification

Transcripts shorter than 200 bp in length were filtered out, number of exons ≥2, expression levels FPKM≥0.5 were retained. Then, the transcripts were identified as lncRNAs or mRNAs by using the software programs CPC [46], and the Pfam database [47], the RNAs that lacked coding potential were considered to be candidates for lncRNAs and were used in subsequent analyses. The basic features of the obtained lncRNAs were characterized by comparison with mRNAs.

Cuffdiff (Version 2.1.1) was used to calculate fragments per kilo-base of FPKMs of both lncRNAs and coding genes. The FPKMs were computed by summing the FPKMs of transcripts in each gene group. Transcription with P-just < 0.05 were classified as differentially expressed.

### Target gene prediction and enrichment analysis

Potential target genes of the lncRNAs were predicted according to their regulatory patterns, which were divided into *cis*- and *trans*-acting groups. For *cis*-regulator prediction, a lncRNA that was 100 kb up- or down-stream of the coding gene was defined as a co-located lncRNA. Prediction of *trans*-regulators was determined by the expression level; the correlation in expression between lncRNAs and coding genes was evaluated using the Pearson’s correlation coefficient (r > 0.95 or < − 0.95 and *p* < 0.05). The intersection between the two groups were extracted (Additional file 9). To gain insight into the functions of the lncRNAs and their corresponding target genes, GO terms and KEGG pathway enrichment analyses of the lncRNAs were performed. The GO ontologies were assigned using Blast2GO [48], and GO terms with a corrected *P*-value < 0.05 (Correction method, FDR) were considered significantly enriched. KEGG annotation was performed using KOBAS (Version 2.0, http://www.genome.jp/kegg/).

### Systematic cluster analysis, protein–protein interaction and co-expression network construction

Systematic cluster analysis of DE-mRNAs, DE-TFs, and their targeted DE-lncRNAs was performed. For DE-mRNAs, those that were correlated with response to hormone stimulus, carotenoid or ethylene biosynthesis, sugar and main organic acid metabolism were selected. Transcription factors that showed fold change > 2 and FPKM > 30 were selected for the analysis. A heat map was generated using Cluster software (Version 3.0) and visualized using Java Treeview (Version 1.0.4). Protein–PPI analysis was based on the STRING database (http://string-db.org/), and a network was constructed by extracting the target gene list from the database. Target genes were aligned to the selected reference protein sequences using Blastx (Version 2.2.28), and the network was constructed in accordance with the known interaction of the reference species (*Cucumis sativus*). The gene IDs of the selected genes (including DE-TFs) were used to isolate the co-expression relations among target genes and lncRNAs, and the P-P interactions in PPI analysis results, respectively. Then, organized in a spreadsheet (Additional file 10) and constructed a co-expression network using Cytoscape (Version 3.7.0).

### Quantitative real-time PCR of lncRNAs

Ten lncRNAs with only one transcript were selected and quantitative real-time PCR (qRT-PCR) was performed as validation. RNA isolation was performed as already described. Poly (A) Polymerase (Takara Bio, Shiga, Japan) was used to add poly A tails following the manufacturer’s instructions, then reverse transcription was performed using the PrimeScript™ RT reagent Kit with gDNA Eraser (Takara Bio, Shiga, Japan). qRT-PCR was performed using SYBR® Premix Ex Taq™ II (Takara Bio, Shiga, Japan) on a BioRad CFX96 instrument with default parameters. Relative gene expression levels were normalized to *GAPDH* and calculated using the 2^−ΔΔCt^ method. Primers used for the validation experiments are shown in Table 2.

**Table 2:** Primers used for quantitative real-time PCR

Gene ID	Forward Primer	Reverse Primer
LNC_000401	AGGAGCCGAATGAAACCAAAG	GAACCCGCATCGTTAGCTTG
LNC_000454	GACACTGAACCACAGATTCCACA	GTTTCGTGTGTTGTTGCTCTGAC
LNC_000636	CAGTGACATACGATGATGGTTGG	CAGTCACATACATCATAAGTCCATCAG
LNC_000987	CATCTTTTCCTTTTCCCCTTTGT	TGATTTGAGGATTCTTGTGGTGG
LNC_001286	AACTTCACATCTCTTTCATCGCA	GGACAAACGCAACGTCTTCAAC
LNC_001730	CTTTCTTTACTCCTCAAACTCCG	GTAGAGGATGAGTTGGCGGC
LNC_002015	GGGAAATGTGTAGAAGAAGCAGT	ATCAATGGACTCTCTATCCTCTCTT
LNC_002556	TCCAAAGTCAAGAGGGAAATAGCC	CTAAGCCCACCTCCTGGTTGTC
LNC_002609	GGAACCAAATAGTTGTGGATGTG	ATGGTGATTGTTTGCTCCTGTC
LNC_000141	TTCCGATTAAATCCTCTTGTTTGT	TTCTCGTCTCCATTCTTCGTCAC
*GAPDH*	CGTGTTCCTACCGTTGATGTCTCT	TCAGTGTACCCCAAAATTCCCTTC

## Additional files

Additional file 1: Details of lncRNAs. (XLSX 275 kb)

Additional file 2: Cis- and Trans-interaction between lncRNAs and mRNAs. (XLSX 20120 kb)

Additional file 3: The GO terms of co-located target genes. (XLS 34 kb)

Additional file 4: Top 20 enriched KEGG pathways of co-located target genes. (XLS 33 kb)

Additional file 5: Enriched cis-target genes in plant hormone signal transduction and Carotenoid biosynthesis. (XLS 191 kb)

Additional file 6: GO terms of co-expressed genes. (XLS 2482 kb)

Additional file 7: Top 20 enriched KEGG pathways of co-expressed target genes. (XLS 63 kb)

Additional file 8: Gene ID of the genes were discussed. (XLSX 31 kb)

Additional file 9: The intersection between co-location and co-expression groups. (XLSX 60 kb)

Additional file 10: Details of the co-expression networks. (XLSX 551 kb)

Additional file 11: **Figure S1.** Clustering analysis of all the differentially expressed lncRNAs. (PDF 149 kb)

Additional file 12: **Figure S2.** The results of the RT-qPCR confirmed the expression patterns of the selected lncRNAs were consistent with the expression levels calculated from the RNA-seq data. (JPG 200 kb)

## Data Availability

The data has been submitting to the SRA (https://www.ncbi.nlm.nih.gov/sra), SRA accession: PRJNA543288.

## Abbreviations

ACO: 1-amino-cyclopropane-1-carboxylate oxidase
AGL1: Agamous-like 1
CPC: Coding Potential Calculator
DAA: Days after anthesis
DE-lncRNAs: Differentially expressed lncRNAs
GO: Gene Ontology
IAA: Auxin indole-3-acetic acid
lncRNAs: Long ncRNAs
ncRNAs: Non-coding RNAs
PPI: Protein interactions
PSY: Phytoene synthase
RIN: Ripening inhibitor
sncRNAs: Small ncRNAs
SUSY: Sucrose synthases Y
TF: Transcription factor
