# Supplementary figures and images for: Genome-wide identification and characterization of long non-coding RNAs involved in fruit ripening and the climacteric in Cucumis melo
Source: BMC Plant Biol. 2019 Aug 22;19:369. doi: 10.1186/s12870-019-1942-4 (PMC6704668; doi:10.1186/s12870-019-1942-4)

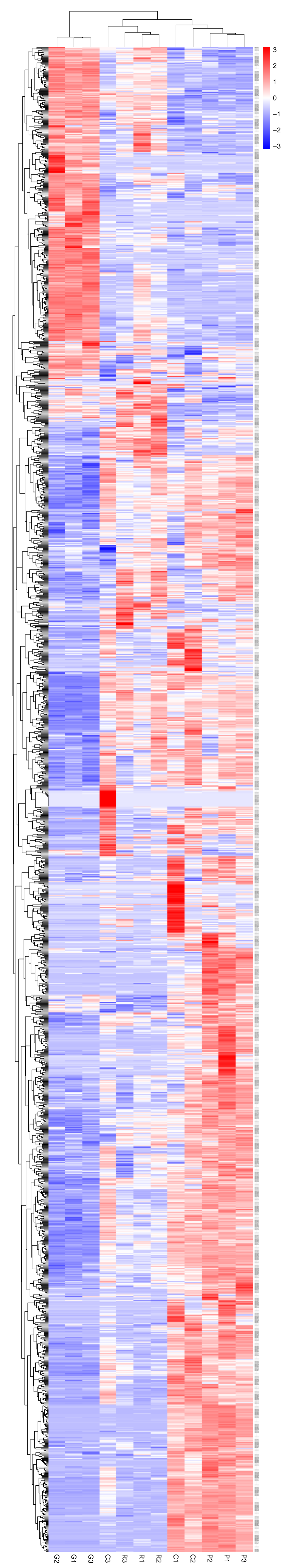

Supplement: Supplementary file 11 — Figure S1. Clustering analysis of all the differentially expressed lncRNAs. (PDF 149 kb) [file 12870_2019_1942_MOESM11_ESM.pdf]

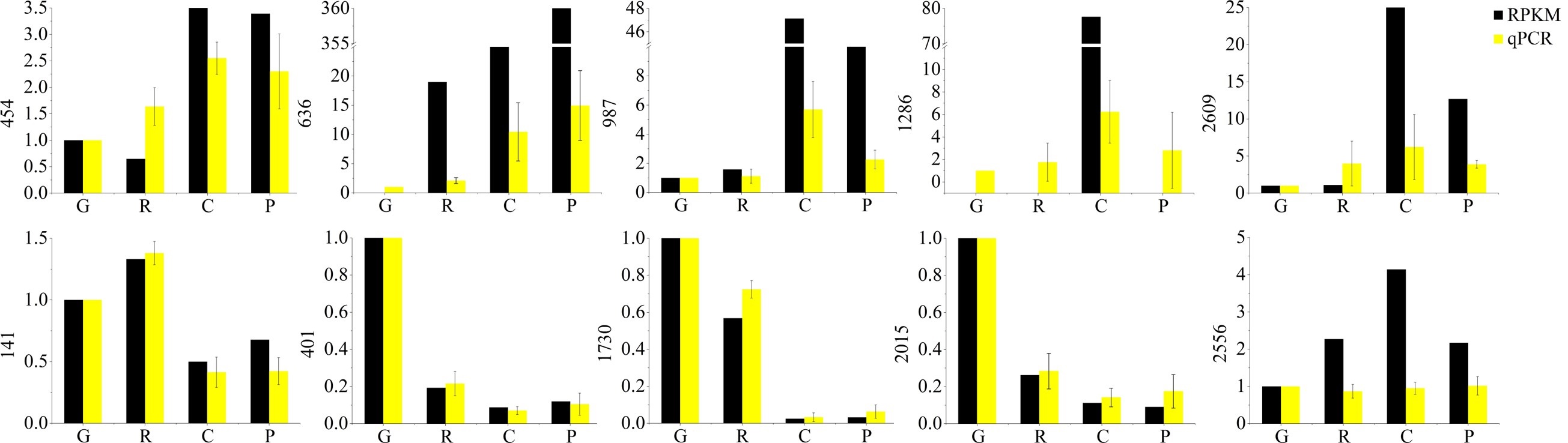

Supplement: Supplementary file 12 — Figure S2. The results of the RT-qPCR confirmed the expression patterns of the selected lncRNAs were consistent with the expression levels calculated from the RNA-seq data. (JPG 200 kb) [file 12870_2019_1942_MOESM12_ESM.jpg]
